# Bilateral septic arthritis of the temporo mandibular joint: case report

**DOI:** 10.11604/pamj.2016.25.100.7943

**Published:** 2016-10-20

**Authors:** Samia Ayachi, Zouha Mziou, Ramzi Moatemri, Habib Khochtali

**Affiliations:** 1 Department of Oral and Maxillofacial Surgery, Sahloul Hospital, Sousse, Tunisia

**Keywords:** Septic arthritis, temporo-mandibular joint, infection, adult

## Abstract

Septic arthritis of the temporo-mandibular joint (TMJ) is a rare disease that has been reported infrequently. To the best of the authors' knowledge, only one case of bilateral TMJ septic arthritis has been reported. The contamination may result from direct extension of adjacent infection (dental or ENT), from hematogenous spread of blood-borne organisms or from direct inoculation. The most common presenting are trismus and pain, although swelling, tenderness and erythema have also been described. In addition, patients may develop fever, regional lymphadenopathy and malocclusion. Through a successively bilateral case of TMJ arthritis, without obvious portal of entry of the bacteria, we will analyze characteristics and treatment of this disease.

## Introduction

Septic arthritis of the temporomandibular joint (TMJ) has rarely been reported [1]. This condition is mainly arises from direct extension of head and neck infection or occasionally by hematogenous dissemination [2]. The most common pathogen isolated in septic arthritis of TMJ is S aureus followed by Neisseria and streptococcus [2, 3]. Clinical manifestations include pain, swelling in the TMJ, trismus and malocclusion. Symptoms are often not as typical as they were previously, owing to the widespread use of antibiotics [1, 2]. Timely diagnosis with early treatment is the most significant predictor of a successful outcome [3]. Through a successively bilateral case of arthritis, without obvious portal of entry of the bacteria, we review literature and we will analyze characteristics and treatment of this topic.

## Patient and observation

**Figure 1:**
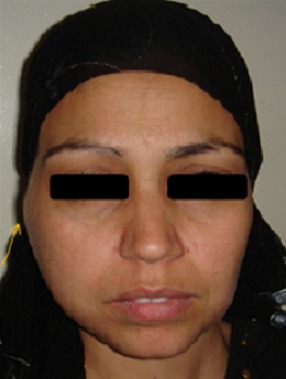
Mild preauricular swelling on the right side

**Figure 2:**
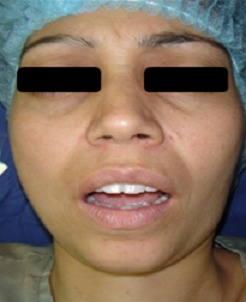
Maximum mouth opening of 10 mm

**Figure 3:**
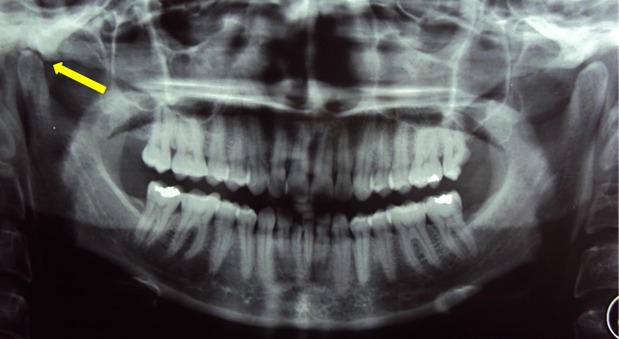
Orthopantomogram showing pinching space of the right TMJ

**Figure 4:**
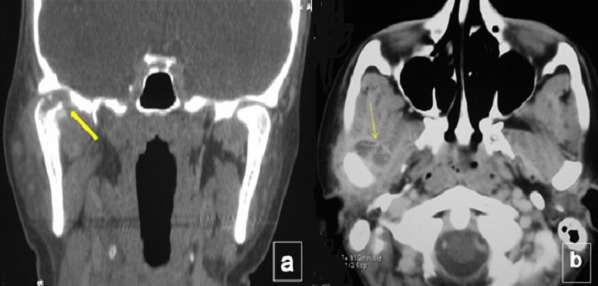
A) CT scan revealing osteolysis of the right mandibular condyle; B) two abscesses in the right infratemporal fossa

**Figure 5:**
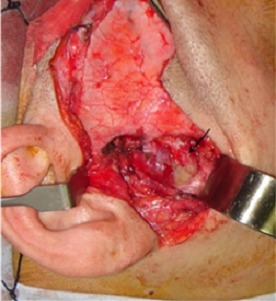
Peroperative view showing abcesses (arrow)

**Figure 6:**
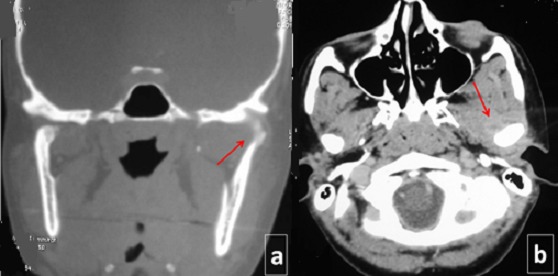
A) CT scan, 1 year later, showing osteolysis of the left condyle; B) without reactivation in the other side and abcesses in soft tissue

An otherwise healthy, 30 year old woman presented to the department of Maxillo-facial surgery complaining of right pre-auricular pain associated with trismus of one month's duration. She had been treated with amoxicilline orally further 10 days with no improvement. The clinical examination revealed mild pre-auricular swelling on the right side (Figure 1) and reduced, painful mouth opening (10 mm) (Figure 2). No adjacent infections (ENT or dental) have been found. The patient presented low-grade pyrexia of 37.4^°^C and her vital signs were normal. Hematological values were as follows: hematocrit: 36.6%; hemoglobin: 12.10 g/100 ml; red blood cell count: 419-104/mm3; white blood cell count: 8360/mm^3^ with 85% polymorpho-nuclear cells. Orthopantomography has showed pinching space of the right TMJ (Figure 3). A computed tomography (CT) scan has objectified osteolysis of the right mandibular condyle and showed a diffuse soft-tissue swelling involving the pre-auricular region with two abscesses in the right infra-temporal fossa (Figure 4). The patient was operated under general anesthesia. A pre-auricular incision with a temporal extension was used. A resection of the lytic condyle, drainage of abcesses and extensive washing of the joint cavity were performed (Figure 5). Culture of the purulent fluid was negative. The histopathologic examination concluded to non-specific chronic active osteomyelitis. After the surgery drainage, she improved immediately and regained full movement of the mandible 5 days later. Amoxicillin and clavulanic acid were continued intravenously for one week, followed by a further 3 weeks given orally. CT scan at 3 months objectified rearrangements without signs of reactivation. One year later, the patient presented the same clinical symptoms on the other side (left) with pre-auricular swelling and painful trismus. Osteoarthritis of the left TMJ was easily diagnosed. CT scan showed a necrosis of left mandibular condyle and abscesses in infra-temporal fossa with no reactivation of the other side (Figure 6). Condylar resection and lavage of condylar cavity were done by a pre-auricular incision. Intravenous antibiotic treatment was started with Amoxicillin and clavulanic acid 1g 3 times a day and changed to oral form during 3 weeks. At 1 month follow-up the facial swelling and tenderness had disappeared and mandibular range of motion improved to 40 mm. The subsequent follow-up examinations showed no further complaints with a decline of 36 months.

## Discussion

Septic arthritis of the temporo-mandibular joint (TMJ) has been reported infrequently [1]. To the best of the authors' knowledge, only one case of bilateral TMJ septic arthritis has been reported [2]. This small number of reported cases may be the result of misdiagnosis or underreporting. Several authors have suggested that a significant percentage of cases of mandibular complications such as ankylosis and abnormal growth, may have resulted from failure to diagnose TMJ septic arthritis [3, 4]. Susceptible patients are those with diabetes, systemic lupus erythematous, rheumatic arthritis, or immunosuppressive diseases [5]. The contamination may result from [6, 7]: extension of adjacent infection in soft tissues or bone (dental or ENT); hematogenous spread of bacteria from distant infection or intravenous drug use; direct inoculation by penetration of the joint space traumatically or intra-operatively. Recent studies have shown that micro-organisms are most commonly seeded hematogenously, but the original infectious sites were often occult. Staphylococcus aureus is the most commonly isolated organism from patients with septic arthritis of TMJ, although Streptococcus species, Neisseria and Haemophilus influenza have also been reported in the literature [6, 8, 9]. The most common presenting are trismus and pain, although swelling, tenderness and erythema have also been described as did our patient. In addition, patients may develop fever, regional lymphadenopathy and malocclusion [1, 2, 9]. In many instances, patients may not have a classic presentation and symptoms may be confused with other diseases, such as cellulitis, rheumatic disease, condylar hypertrophy or synovial chondromatosis [1, 3]. Imaging studies are helpful for diagnosis [7]. Joint space widening is generally demonstrated in panoramic in the acute stage [2].

CT scan is advantageous in chronic stage because it can show the bony changes earlier in addition of peri-articular soft tissues and identify infectious osteoarthritis [8, 9]. Erosive changes were seen on the CT of our patient when she had been symptomatic only 2 weeks. The magnetic resonance imaging is recommended because its sensitivity for the early detection of increased joint effusion [2, 5]. Laboratory examinations are also helpful for the diagnosis if inflammatory signs are found but sometimes they can be normal because of the use of antibiotics [2]. Although the diagnosis of septic arthritis of the TMJ is ideally made by the identification of organisms in the aspirated fluid [2, 3, 10]. Therefore other evidence, such as clinical presentation, radiographic appearance, laboratory values, response to antibiotics and exclusion of other disease may provide critical informations. Treatment should be initiated promptly to avoid irreversible complications such as abscess or necrosis of the condyle [8]. The treatment of osteoarthritis consists primarily of rest therapy (restricting jaw movements), medical treatment (analgesics, antinflammatories), splint therapy or minimally invasive therapy. Antibiotic treatment must be continued for as long as possible, a total of 30 days therapy has been recommended [2, 3, 8]. Arthroscopy, being a minimally invasive procedure, should be preferred to open surgery when possible as the initial treatment and not only after needle aspiration failure[3, 10]. Surgical drainage has been suggested if abscess formation is present, if bony changes are radiographically evident or when antimicrobial therapy alone has failed to produce resolution of infection [3, 8]. Our patient was operated in front of the presence of abscess and peri-articular osteolysis condyle. The bacteriological examination was negative because of use of antibiotics. Infected joints usually become sterile 48 to 72 h after drainage [2]. Physical therapy is necessary to improve mandibular range of motion and deviation of opening movement. Moreover early rehabilitation exercises are important to ovoid complications such as fibrosis and ankylosis [2, 3].

## Conclusion

Septic arthritis of the TMJ is a rarely reported entity whose occurrence might be more common if clinicians were aware of its possibility. Early diagnosis and treatment are essential to avoid possible sequelae and complications such as dissemination of infection, joint dysfunction, growth disturbances, fibrosis, and ankylosis.
